# A multi-method approach to characterising dynamic human–shark interactions at a remote oceanic island

**DOI:** 10.1038/s41598-026-46394-0

**Published:** 2026-04-13

**Authors:** Lucy Clarke, Claire Collins, Polly Burns, Laura Chapel, David J. Curnick, Philip D. Doherty, Olivia Goodchild, Matthew Gollock, Nigel E. Hussey, Esben Lomholt, Daniel Simpson, Tiffany Simpson, Nicola Weber, Sam B. Weber

**Affiliations:** 1 Faculty for Environment, Science and the Economy, Centre for Ecology & Conservation, University of Exeter, Penryn, Cornwall, TR10 9FE UK; 2 Institute of Zoology, Zoological Society of London, Regent’s Park, London, NW1 4RY UK; 3 Department of Environment and Geography, University of York, Wentworth Way, York, YO10 5DD UK; 4 Department of Genetics, Evolution and Environment, University College London, Gower Street, London, WC1E 6BT UK; 5 Conservation and Policy, Zoological Society of London, Regent’s Park, London, NW1 4RY UK; 6 Department of Integrative Biology, University of Windsor, 401 Sunset Avenue, Windsor, ON N9B 3P4 Canada; 7 Ascension Island Government, Conservation & Fisheries Directorate, Georgetown, Ascension Island ASCN 1ZZ UK

**Keywords:** Galapagos shark (*Carcharhinus galapagensis*), Human-wildlife conflict, Sentiment analysis, Marine protected area, Social media, Ecology, Ecology, Ocean sciences

## Abstract

Negative human-wildlife interactions (HWI) pose a growing global conservation challenge, yet marine contexts remain underexplored relative to terrestrial systems. Sharks, as an ecologically critical but socially polarising group, exemplify these challenges and current monitoring frameworks often overlook the dynamic and complex nature of human–shark interactions (HSI). Here, we adopt a multi-method approach to characterise HSI at Ascension Island; a remote oceanic island surrounded by one of the world’s largest marine protected areas. By synthesising data from social media (*n* = 135 posts), semi-structured interviews (*n* = 26 interviewees), an online survey (*n* = 14 responses), archival records (*n* = 59 accounts), and remote camera footage (*n* = 325 days monitored), we (1) quantify spatiotemporal variation in HSI, (2) examine how the nature of HSI has changed, and (3) assess changes in local sentiment. Findings reveal fluctuations in the frequency of HSI across multiple temporal scales, from intra- and inter-annual variation to broader decadal oscillations. Collectively, the results suggest a decline in sightings of sharks during the late 20th century, followed by a recent resurgence preceding two shark bites in 2017. Temporal variation in HSI was associated with periods of heightened inshore shark presence and rapid behavioural shifts (e.g. increased aggression), whilst sentiment analysis highlights diverse and shifting views toward sharks, suggesting possible resilience or evolving acceptance within the community. This study highlights the value of a multi-method approach to better understand spatiotemporal dynamics of HSI and support adaptive management to foster coexistence. We underscore the need for monitoring approaches that account for both social and ecological dimensions of HSI and call for further research into the environmental drivers of observed variation in HSI.

**Supplementary Information:**

The online version contains supplementary material available at 10.1038/s41598-026-46394-0.

## Introduction

As wild habitats are increasingly encroached upon, humans and wildlife are forced to share limited space and resources, with evidence suggesting a corresponding increase in the frequency of human-wildlife interactions (HWI)^1^. Repeated negative HWI can escalate to human-wildlife conflict (HWC)^2^, defined as ‘wildlife posing a direct or recurrent threat, through its behaviour or presence, to human livelihoods and/or safety’^3^. HWC represents a significant challenge to conservation as increasing antagonism towards the species involved can undermine support and compliance with conservation initiatives threatening their long-term effectiveness Further, impacted communities may engage in direct mortality or advocate for lethal population control including the removal of perceived “problem animals”^2,4^. Crucially, HWC’s are not solely determined by the frequency of negative interactions, but are driven by a complex interplay of environmental, ecological, and anthropogenic factors^5,6^ and are therefore highly dynamic across space and time^1,7^. Understanding these complexities is critical for informing public discourse^8^, predicting future interactions, and guiding the development of effective management solutions^6,9^.

Monitoring the frequency and nature of HWI is fundamental to understanding HWC. However, whilst the monitoring of HWI is becoming increasingly commonplace across terrestrial systems^1,10,11^, it remains understudied within marine environments^12^. Methods for monitoring HWI include direct observation (e.g. using uncrewed aerial vehicles^13^ and, remote cameras^14,15^, risk mapping (e.g. wildlife tracking and distribution modelling^16,17^, citizen science initiatives (including voluntary community reporting schemes^18,19^ and post hoc stakeholder interviews^20–22^. However, whilst these approaches have improved collective understanding of HWI, they face limitations when individually applied. For example, voluntary reporting schemes rely on high levels of engagement from affected parties and trust in the relevant management authorities, which can break down in cases of HWC^23^. Monitoring of HWI through direct observations can yield more quantitative data but often suffers from limitations related to spatiotemporal coverage^24^, whilst post-hoc interviews are reliant on participant recollection and may be subject to recollection bias generated by HWC^7,25^.

Importantly, limited integration of historical baselines amongst more recent data sources constrains the assessment of temporal trends in HWI and limits insight into relative change^26,27^. Approaches that synthesise multiple data streams and integrate novel data sources are therefore critical for developing a holistic understanding of contemporary HWI^1,23^. Archival records, for example, serve as valuable historical benchmarks for interpreting contemporary ecological trends^28^, but have been little used to monitor the spatiotemporal dynamics of HWI. Similarly, social media platforms are a vast, yet currently underutilised repository of information^29^. The abundance of user-generated content, combining visual records of wildlife interactions with location and timestamp metadata, alongside textual reflections of public sentiment, provides a novel resource for monitoring ecological phenomena^30,31^. However, its application in tracking trends in the occurrence and characteristics of marine HWI remains largely limited. Both archival and digital sources can also possess inherent biases that disproportionately reflect negative or sensational encounters, whilst overlooking everyday occurrences. On social media, this can be further amplified by algorithms that reinforce public perceptions of increasing conflict. Acknowledging these biases is essential and supports the strength of a multimodal approach through triangulation of data streams to aid in distinguishing ecological trends from artefacts of increased reporting.

Human–shark interactions (HSI) are among the most widely reported examples of HWI in the marine environment^32^. Despite their relatively low occurrence^33–35^ shark bites dominate public discourse and media narratives of HSI^36^ due to the evocation of strong emotional responses^8,37,38^. This emphasis is mirrored in global monitoring systems such as the International Shark Attack File (ISAF), which catalogues shark bites. However, HSI are not limited to bites, but can encompass a broad spectrum of interactions^32^ including positive interactions, such as ecotourism^39^ and cultural or spiritual associations with sharks^40,41^, reflecting the endemic value attributed to these species amongst local communities^42^. Conversely, HSI also encompasses negative interactions, such as commercial and recreational fisheries catch depredation^43,44^. An increase in negative HSI can prompt human-human conflict regarding the implementation of shark conservation initiatives^45^, particularly in regions where livelihoods or ocean-user safety perceptions are impacted^46–48^. Given the worsening conservation status of many shark species^49^ and the potential of negative HSI to undermine protection efforts^45^, it is critical to monitor HSI trends and identify drivers of increasing interactions. However, by focusing predominantly on shark bites, current reporting frameworks, such as ISAF, overlook the broader ecological, cultural, and socio-economic dimensions of HSI. Moreover, the frequency and nature of HSI often demonstrates high levels of spatiotemporal variation^50–53^. Consequently no single data source can encompass the dynamic and multifaceted nature of HSI, underscoring the need for integrated, interdisciplinary approaches that better capture this complexity.

**Fig. 1:**
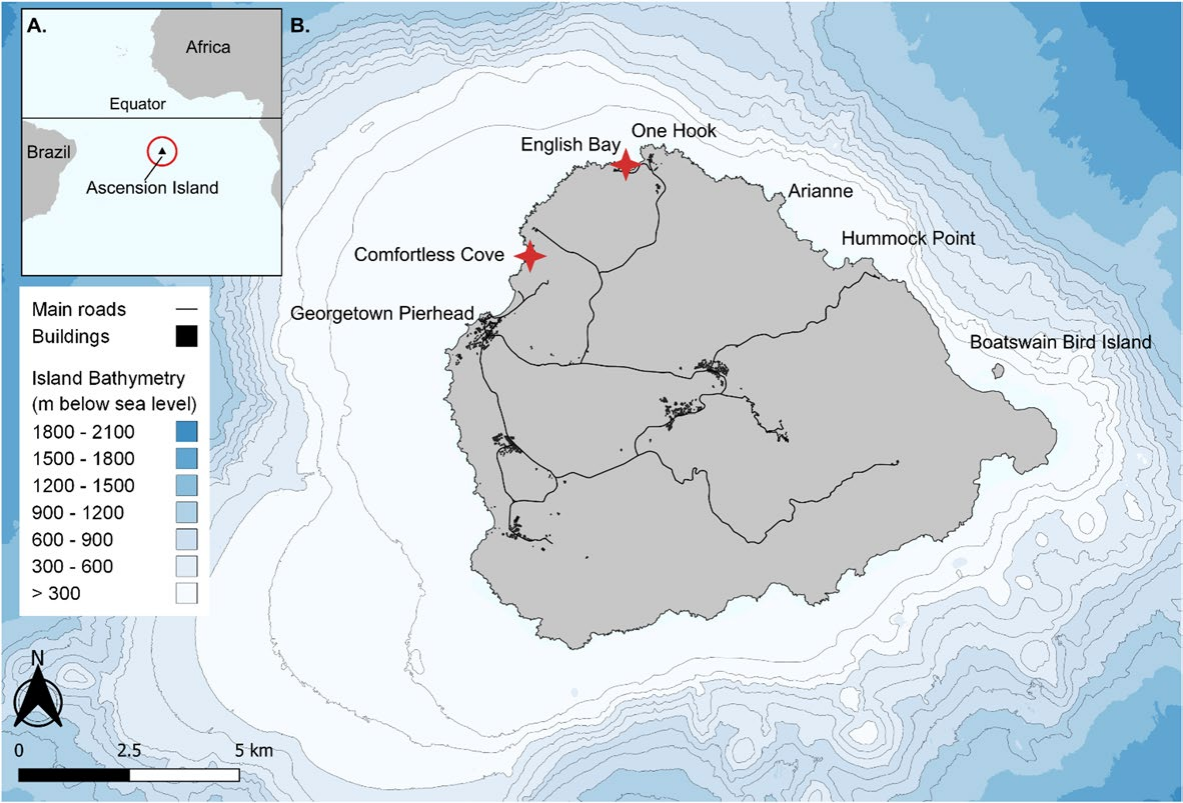
(**A**) Location of Ascension Island within the Atlantic Ocean, (**B**) Study site with island bathymetry (blue shading), and key locations of shark sightings labelled. Red crosses denote the two primary bathing spots on-island, Comfortless Cove and English Bay; the two sharks bites of 2017 occurred at English Bay. A remote time lapse camera is located at the Georgetown Pierhead. Figure was created using QGIS version 3.34.15 (www.qgis.org).

In this paper, we adopt a multi-method approach to characterise dynamics of HSI at Ascension Island, a remote) Overseas Territory of the United Kingdom (UK), located in the tropical South Atlantic (Fig. 1). As a recently established large-scale MPA with limited standardised monitoring, Ascension Island provides an ideal case study to demonstrate how the triangulation of diverse data sources - such as archival records and social media uploads - can be synthesised to reconstruct HSI trends in remote, data-poor environments. Ascension Island has recently garnered global media attention following two non-fatal, but serious, shark bites occurring in quick succession in April and July of 2017^54,55^. Although unconfirmed, the bites have been widely attributed to Galapagos sharks (*Carcharhinus galapagensis*) due to their heightened presence at inshore coastal locations preceding the bites, alongside an increase in interactions with ocean users and high levels of fisheries depredation being reported^56,57^. Galapagos sharks are historically responsible for very few unprovoked incidents globally^35^, making the cluster of events at Ascension Island an anomaly and underscores the importance of situating increases in HSI within a broader historical and ecological framework. In the absence of contextual baselines, apparent increases in negative HSI can fuel speculation regarding drivers and can diminish public support for shark conservation, particularly where protective measures are perceived to conflict with human safety^58–60^.

Here, we aim to characterise dynamics of HSI over multiple timescales to provide a quantitative basis for public discourse and future research into underlying drivers of interactions. Specifically, we examine how the frequency and spatial distribution of HSI has varied over short (monthly), medium (annual), and long-term (decadal) timescales. We then assess whether the nature of these interactions has shifted over time and explore concurrent changes in human sentiment toward sharks. Drawing on multiple complementary data sources, we profile HSI at Ascension Island and demonstrate the broader applicability of this integrative framework for monitoring human–wildlife interactions across alternative geographies and taxa.

## Results

### How has the frequency of human–shark interactions varied over time?

#### Short-term (intra-annual) variation

**Fig. 2:**
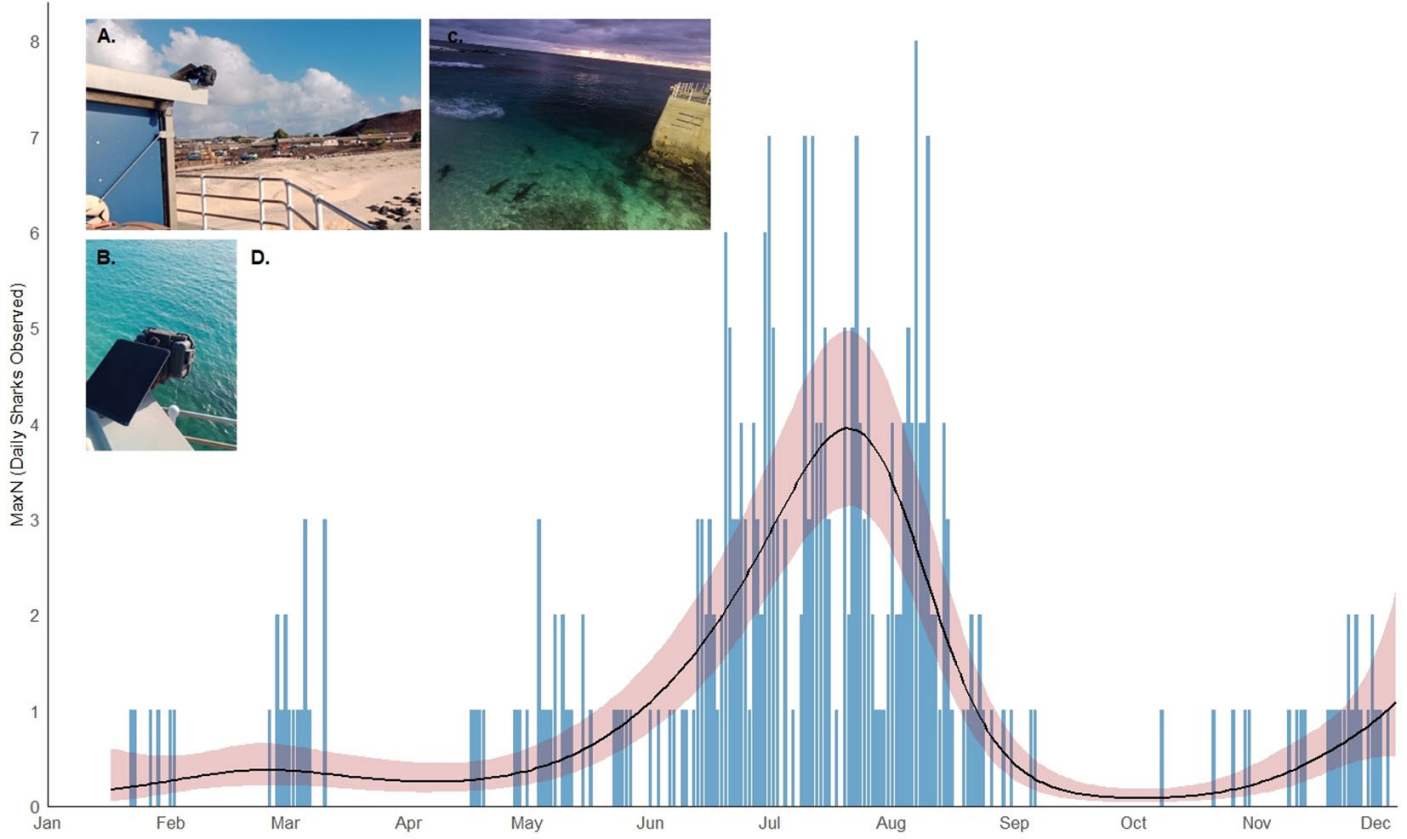
(**A**) The position and (**B**) set-up of the remote timelapse camera installed at the island pierhead near Georgetown - programmed to take a still image every 10 min for 24 h a day from January 2024 to December 2024. (**C**) An example of an image taken and processed for analysis (image shown taken on 10/07/2024 20:10:50) (**D**) Intra-annual variation in inshore shark presence at Georgetown Pierhead between January and December 2024 based on daily maximum number of sharks observed (MaxN) using a remote time-lapse camera. The black line and shaded ribbon denote the predicted MaxN and associated 95% confidence intervals from the fitted generalized additive model (GAM).

At the finest temporal scale, analysis of photographic data (*n* = 34,810 images) collected by a remote timelapse camera located at Georgetown Pierhead revealed significant short-term variation in nearshore shark activity from January to December 2024 (Fig. 2). The mean daily MaxN of sharks detected by the camera (determined by calculating the maximum number of sharks observed in a single image for each day in the timeseries), was relatively low for the first six months of monitoring but peaked sharply across July and August, before declining to a low background level (GAM, χ² = 231.7, *p* < 0.001). Mean MaxN peaked at 4 sharks per day in July, with a maximum of 8 individuals observed in a single image on 7th August 2024. Shark activity at the Pierhead also varied significantly over the diel cycle, with the proportion of images containing sharks increasingly sharply at night and peaking at 21:00–23:00 (GAM, χ² = 1027, *p* < 0.001; SI 2). Sharks were observed in just 0.51% (*n* = 97) images taken in the day compared to 7.0% (*n* = 1103) of images captured at night. Although image clarity was generally not sufficient to verify species identity, firsthand reports from competent observers suggest that most/all individuals present around the Pierhead during this time were Galapagos sharks.

To investigate if the intra-annual variation recorded by the Pierhead camera in Georgetown represents consistent seasonality in HSI, monthly social media uploads containing either video or photographic evidence of inshore shark activity (*n* = 125) were also analysed for the period 2010–2024. A significant weakly bimodal trend was detected in the proportion of annual social media uploads occurring in each month (binomial GAM, χ² = 19.14, *p* < 0.01; SI 3), with peaks in March and August, although posts occurred across the year. There was also a lack of consensus amongst SSI and online survey respondents regarding seasonality of shark interactions. One SSI respondent who had been living on Island for over 25 years, stated that “*I don’t think there is any particular season for the sharks*” whilst only 45% (*n* = 18) of SSI and online survey respondents agreed that seasonality had an influence on the levels of HSI.

#### Medium-term (inter-annual) variation

**Fig. 3:**
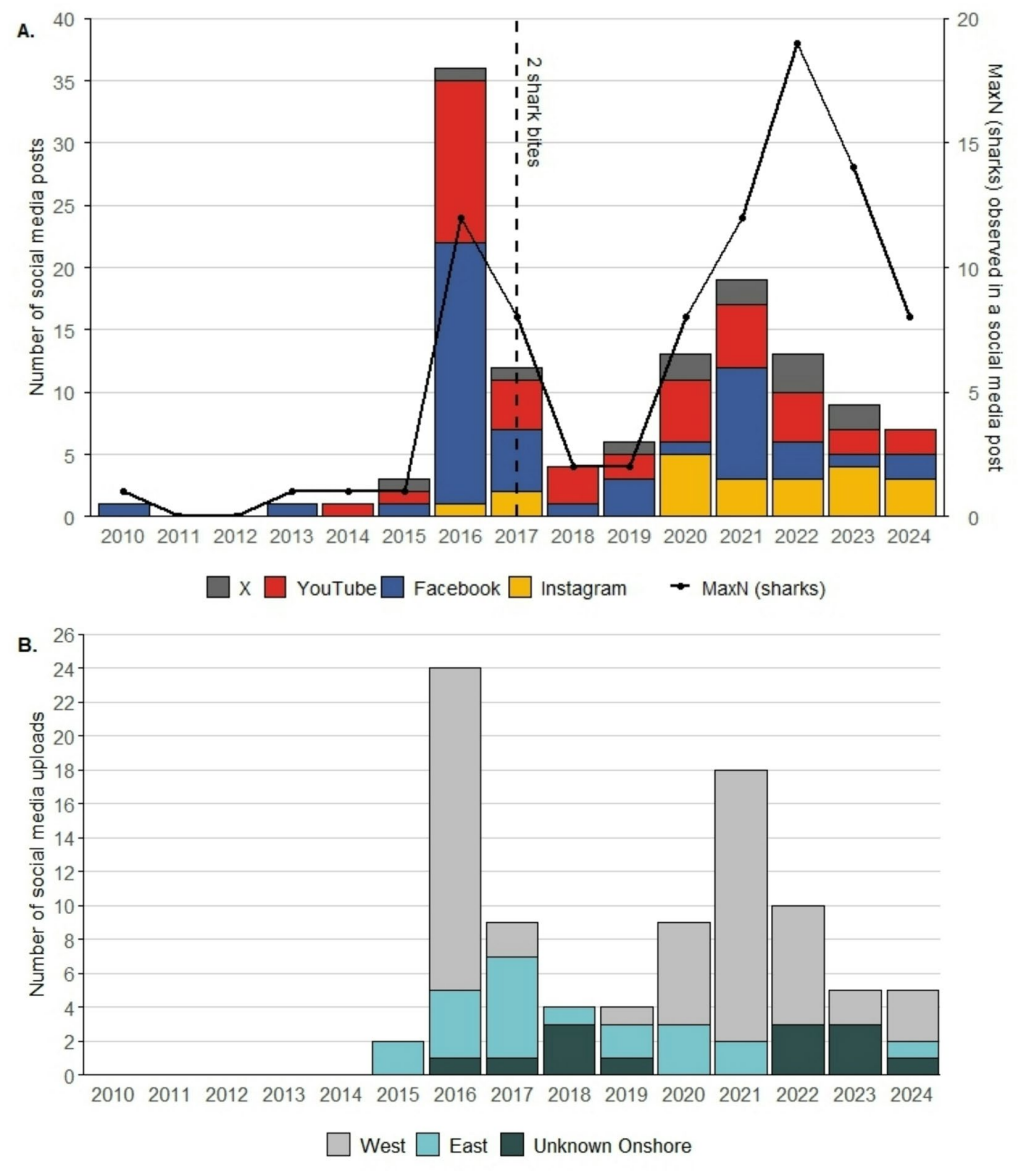
Spatio-temporal trends in social media uploads featuring photographic or video footage of Galapagos and/or silky sharks at Ascension Island (*n* = 125; 2010–2024). (**A**) Combined count of annual uploads across major platforms (X (formerly Twitter), YouTube, Facebook, Instagram). The vertical dotted black line marks two shark bites in 2017, while the solid black line represents the maximum number of individuals shown in any single upload for each year. (**B**) Breakdown of upload locations over time, categorised as inshore West (Pierhead, Comfortless Cove, One Hook, English Bay), inshore East (Hummock Point, Arianne, Boatswain Bird Island), and Unknown inshore (where location could not be determined).

Analysis of social media posts (*n* = 125) collated from four leading platforms (Youtube, Facebook, X, and Instagram; See SI 4 for breakdown) also suggest significant inter-annual variation in the frequency of HSI across the period for which data were available (2010–2024; Fig. 3). Two pronounced peaks were apparent in both the annual number of uploads (GAM; χ² =15.63, edf = 3.06, *p* < 0.001) and the annual MaxN of sharks documented in a single post (GAM, χ² =17.63, edf = 1.77, *p* < 0.001), one in 2016 preceding the two bites on ocean users at English Bay in 2017, and a second in 2021 (Fig. 3A). Whilst the number of social media posts featuring sharks peaked in 2016 (*n* = 36), the maximum number of sharks observed was highest during 2021, with 19 individuals captured in a single image taken from Georgetown Pierhead in a post from July 2022 (Fig. 3A). Analysis of posts that could be georeferenced shows that temporal trends were largely driven by an increase in numbers of sightings from inshore areas on the more populated west coast (Fig. 3B; see SI 5 for granular breakdown by location). Posts classified as inshore accounted for 67% of the 2016 peaks and 95% during the 2021 peak. Specifically, the Georgetown Pierhead emerged as a site of consistent and repeated inshore shark presence and accounted for 57% (*n* = 51) of inshore posts.

#### Long-term (decadal) variation

**Fig. 4:**
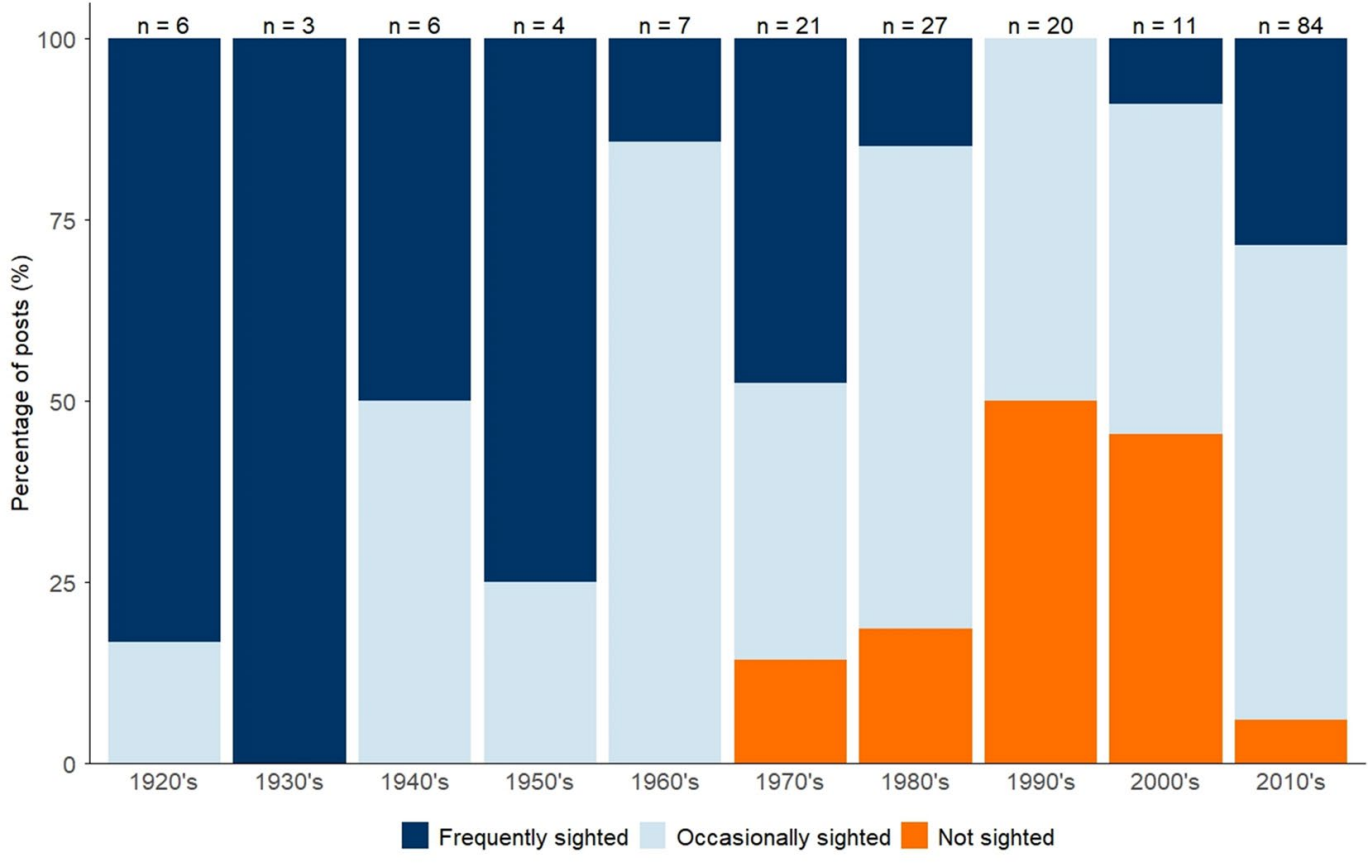
Decadal trends in perceived shark abundance from qualitative historical accounts, social media posts and comment trails, SSIs and online survey responses (*n* = 189), categorised by reported frequency of sightings: frequently sighted, occasionally sighted, and not sighted.

To determine how HSI at Ascension Island has varied across longer timescales, we used qualitative (textual) data drawn from social media posts, social media comment trails, SSIs, survey responses and archival quotes to summarise changes in perceived shark abundance by decade. For the purposes of this analysis, we retained any quote that explicitly referenced shark abundance within a clearly defined timeframe, yielding a total of 189 sources spanning the period 1920–2024.Analysis of data from all sources indicates a significant decadal trend in the frequency of HSI, with a notable decline in encounters in the late 20th century and early 21st century, followed by a more recent increase (Fig. 4). The proportion of quotes that classified sharks as being “frequently sighted” decreased significantly between 1970 and 2010 (GAM, *p* = 0.012, see SI 1 for model spec) before increasing again in 2010–2020.Notably, no sources from 1990–2000 referred to frequent sightings of sharks, and 50% (n = 10) of accounts in this decade explicitly noted that sharks were not present and/or not sighted. In contrast, across all data sources, 29% of posts from 2010–2020 (n = 24) classified sharks as being ‘frequently sighted,’ while only 6% (n = 5) reported an absence of sightings. It is worth noting that all ‘not sighted’ reports from this period occurred between 2010 and 2015, which is consistent with recent trends observed in social media uploads (Fig. 3).

### How has the nature of human–shark interactions changed over time?

In addition to reconstructing long-term trends in the frequency of HSI, textual data from social media, SSIs, online questionnaires and archival sources also provided insights into the shifting nature of these interactions. Following a thematic analysis approach^61^, qualitative data were coded against a thematic framework (SI 5) to identify key themes relating to shark behaviour, abundance and spatial and temporal trends. Fisheries catch depredation emerged as a persistent feature of HSI throughout the study period and was recorded at Ascension Island as far back as the early 18th century, where an account from 1715 describes the frequent loss of fishing bait and gear to sharks: “*we were often plagu’d [sic] with young Sharks*,* that wou’d [sic] run away with our Hooks and Lines*.” The behaviours described here are also mirrored in more contemporary sources, with one interview participant stating that, *“I used to go night fishing but it is really not worth it anymore…I have lost 6 to 7 lures sometimes over 2 to 3 hours*,* and it’s just too expensive*” and shared language across fishers referencing sharks as “*The Taxman*.” Almost all (96%, *n* = 25) SSI participants cited depredation of catch as an observed behaviour or noted lost lures as an impact of shark presence. Yet, fluctuations in reported depredation were also apparent with some respondents recalling periods of markedly lower incidence. One SSI participant noted, “*2020 was the worst time for lures*,* they took everything including lures and fish. No sharks in the 90’s and I spearfished the whole time*,” suggesting that whilst depredation is a long-standing pattern at Ascension Island, its intensity and frequency exhibit oscillations across time.

A repeated theme that emerged, particularly during SSIs and historical archives, was apprehension relating to ocean safety due to predation. Archival reports from the 1920s and 1930s document restrictions around entering the water; “*owing to sharks*,* bathing in the open sea is prohibited”* (1923), a theme echoed in mid-century and more recent observations; “…*sea bathing*,* owing to the danger of sharks*,* was only allowed at Comfortless Cove*” (1944), whilst another notes that, “*Bathing. It is*,* at present*,* an offence to swim in the sea without a permit*,* due to the dangerous sea conditions and the presence of sharks*” (1964). However, in accordance with our results indicating relatively lower shark activity in the 1990s and 2000s, these decades were marked by fewer indications of public apprehension regarding ocean recreation. One social media comment notes that, “*Up until recently English Bay has been fine for swimming. It has been used for the twenty plus years I’ve been living here. Just a lot more sharks around at the moment and no one seems to really know why*” (2017).

Concerns regarding ocean recreation are mirrored by observed changes in shark behaviour. Sources reveal perceived variation in shark temperament, with sharks on occasion being described as bolder, more inquisitive, and at times aggressive, with one SSI participant noting that, “*When sharks are about*,* we be on high alert [so we] don’t agitate water*,* rinse with the seawater to wash your hands*,* not sticking your hand over*,* as they will go for anything*” whilst another SSI participant stated, *“there were multiple sharks coming at the boat before*,* that was in 2021… they were literally moving the boat just by bumping it.”* Comments on social media posts also noted observable changes in physical condition, with one post noting that: “*Having spent a lot of time here in the water with them I have noticed they are very slender”* (2017). Social media comments in 2017 also referenced changes to shark posture and movement “*Be aware of the shark’s behaviour. Arching back. Pectoral fins down. Sharp turns and fast movements*” which are often attributed to signs of aggression or warnings of potential shark bites. These explicit remarks of aggressive incidences of HSI also coincide temporally with an observed peak in social media uploads in 2017 (Fig. 3A).

Thematic analysis of archival records, social media, and survey data also indicates that both perceived shark behaviour and inshore presence are subject to rapid, intra-annual shifts, with accounts describing sudden incursions into areas of human-use. One comment from social media noted that, the population “*seemed to explode” (2021) whilst another social media user states that “Being in the water with them in early 2016 and later on - there is a difference in behaviour.”* Multiple accounts also link sudden increases in abundance with heightened aggression, such as a 2017 social media post of at least “*20 big sharks off English Bay*” and saving a tender *“after being alerted by police about a shark biting their boat’s ropes”.* Whilst accounts demonstrate the potential for rapid, intra-annual shifts in shark presence, reports also suggest contrasting temperaments within known periods of shark presence. Archival sources reference the “*docility of the shark*” (1975), with one survey respondent noting that sharks “*were not interested in swimmers”” (1970’s).* This is further reinforced by analysis of social media uploads; Of 18 videos or photographs where users were diving or snorkelling with sharks, 56% (*n* = 10) were classified as a passive interaction, (whereby the shark did not approach), with users noting that “*they never bothered us*,* even with a catch bag!”* Despite these volatile temporal dynamics, 100% of SSI participants (*n* = 26) strongly or slightly agreed that sharks are important for a healthy marine environment, with some accounts also revealing positive HSI, such as text accompanying one social media post: “*Observing Galapagos Sharks during a wall dive at Boatswain Bird Island*” (2016).

### What have been the impacts of interactions on human sentiment towards sharks?

**Fig. 5:**
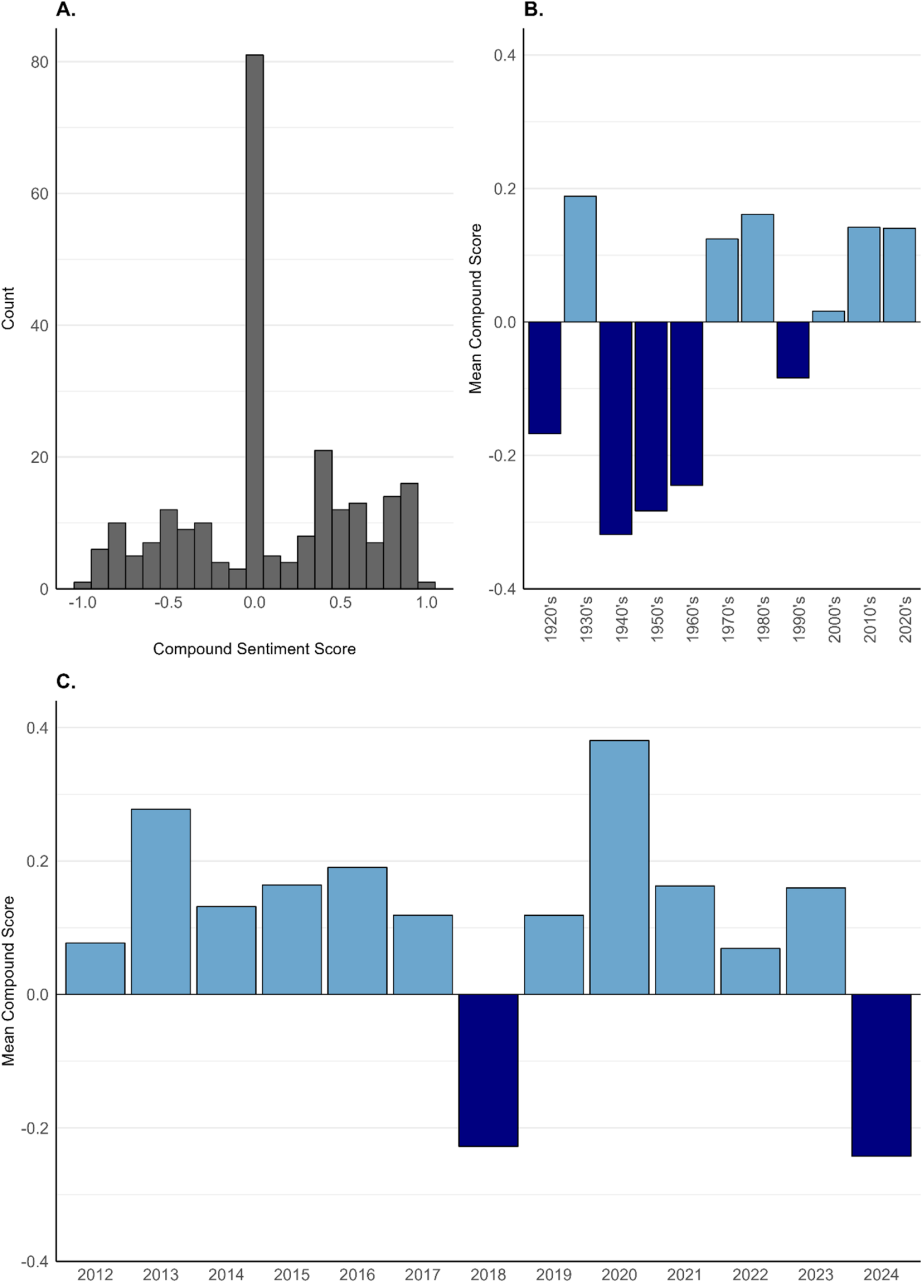
Compound sentiment scores relating to sharks at Ascension Island, calculated as a weighted sum of individual word scores and normalized on a scale from − 1 (negative) to + 1 (positive). (**A**) Histogram of counts of instances of each sentiment score from all quotes (*n* = 249). (**B)** Mean decadal compound sentiment scores from last century (1920–2020, *n* = 236) C. Mean compound sentiment scores in social media era (2012–2024, *n* = 138).

To examine the impact of HSI on human attitudes toward sharks, we processed textual data from social media posts and comment trails, historical archives, and short-form responses from our online survey that refer to a specific timeframe between 1684 and 1920 (*n* = 249). Results indicate a wide diversity of opinion within Island discourse relating to sharks, with 33% (*n* = 81) of passages classified as “Neutral”, 41% (*n* = 101) classified as “Positive”, and 27% (*n* = 67) classified as “Negative” (Fig. 5A).Due to the scarcity of content from pre-1920, data were then truncated to include only passages from the last century (*n* = 236). Decadal variation is evident over the last century, with earlier periods marked by negativity, followed by more recent decades - particularly post-1980 - showing a trend toward positivity (Fig. 5B). Over the last 12 years, a period marked by an explosion in social media use, sentiment has remained largely positive (Fig. 5C). For example, a social media post accompanying images of Galapagos sharks: “*Some of my dive buddies from the last 2 weeks! … we found unusually high numbers making for some superb diving. Ascension Island Nov 16*”. However, the annual resolution of the compound sentiment score also reveals volatile shifts. A marked decline in sentiment is observed in 2018, decreasing from 0.119 in 2017 to − 0.228, which is followed by a rapid rebound in 2019–0.119.

## Discussion

Understanding the complex dynamics of human–shark interactions (HSI) is essential to elucidate underlying drivers and foster coexistence with marine predators, including maintaining long-term public support for marine conservation initiatives. Here, the novel integration of social media, SSIs, an online survey and archival records, represents, to our knowledge, the first study to synthesise publicly available digital content, historical texts, and local perspectives to explore patterns of HSI. Our multi-method approach revealed fluctuations in the frequency and nature of HSI across multiple temporal scales, from short-term intra and inter-annual variation to broader decadal oscillations. We find dynamic patterns of shark presence, with fine-scale spatiotemporal variation in both remote camera footage and social media posts. Thematic analysis highlights a persistent pattern of depredation, recurring accounts of shark aggression at points of perceived increased inshore abundance, and temporally variable shifts in shark behaviour and public sentiment. While historical literature indicates a degree of cyclical trends in abundance of sharks at Ascension Island, our findings suggest that the frequency and nature of HSI has shifted over time and underscore the dynamic nature of HSI at Ascension Island. They also present a scalable, transferable multi-method model that can be adapted to monitor HSI - and potentially predict periods of heightened risk - or can be applied more broadly to HWI in other regions and particularly in data-poor contexts. Interdisciplinary approaches that integrate diverse data sources are especially critical in marine environments, where limited baselines and data scarcity often obscure the emergence of HWC, yet understanding these dynamics is essential for advancing conservation goals.

The frequency of HSI at Ascension Island exhibits variability across multiple temporal scales, suggesting influences from a range of ecological and/or anthropogenic drivers. Over longer, decadal timescales, observed trends in HSI may reflect underlying population-level processes and trends in species abundance, mirroring those observed in other large pelagic predators^62–64^. Long-term, standardised catch-per-unit-effort (CPUE) data from shark control programs, such as those from beach protection nets in KwaZulu-Natal, South Africa^65^, reveal pronounced oscillatory patterns in inferred shark abundance from CPUE with peaks linked to management-driven changes. Similar long-term monitoring in Australia from the Queensland Shark Control Program reveal decadal declines amongst apex shark species^66^. Caution is required when interpreting data from shark control programmes due to non-standardised reporting, often resulting from changes in contractors, gear efficiency, and historical inconsistencies, which can decouple CPUE from true population trends^67^. Despite such limitations, they remain a primary source of data to aid in identifying broad, decadal-scale trends, that can arise through natural predator-prey cycles, climate oscillations^63^(e.g. El Niño–Southern Oscillation & North Atlantic Oscillation), and anthropogenic pressures such as fisheries.

One explanation of the trends we report in this study could be credited, in part, to regional fisheries dynamics, including the historical prevalence of longline fisheries targeting bigeye tuna (*Thunnus obesus*)^68^. The decline of this fishery in the mid-1990s, and its eventual closure within Ascension Island’s EEZ following the designation of the MPA, may have had cascading effects on pelagic predator populations in the wider region^69,70^. Given that the removal of fishing pressure on large pelagic teleost’s may facilitate recovery in shark prey availability, we cannot rule out the potential influence of the longline fishery closure on shark abundance. However, trophic responses are unlikely to manifest over the short temporal scale in which changes in abundance were observed. Recent observed increases in HSI temporally coincide with these protections, including the introduction of legal safeguards for sharks in 2017, leading to perceptions amongst some local inhabitants that there could be a causal link^71^. The designation of the MPA and increased shark protections also highlight how conservation success can prompt human-human conflict, and may be perceived by the local community as directly compromising public safety. However, landings and discard data suggest that Galapagos sharks were rarely (if ever) caught by the commercial longline fleet, which operated mainly in offshore habitats that are unlikely to overlap with the core range of this coastal-pelagic species^70^. Moreover, the abrupt increase in HSI in 2016, preceding the shark bites of 2017 is inconsistent with the species’ slow life-history^72^, making a post-closure population recovery of this magnitude biologically implausible. Nonetheless, this may have reinforced local perceptions of a causal link between conservation action and rising shark encounters. These findings therefore highlight the importance of understanding how conservation success, particularly within MPAs, can intensify human-human conflict. Our results therefore underscore the need to explore how recovering predator populations may reshape risk perceptions and social dynamics in small, tightly connected communities and calls for more research to elucidate ecological and environmental drivers of shark abundance.

Whilst demographic processes may explain long-term trends in perceived shark abundance, they cannot account for the rapid year-to-year and intra-annual variability in HSI that we report from social media uploads and direct camera observations. Instead, rapid fluctuations observed within these datasets are suggestive of an additional layer of short-term environmental variability influencing shark distributions. Recreational fisheries practices, such as discards from shore-based fish processing areas, have been proposed as potential drivers of inshore shark movements and research in similar systems, such as Norfolk Island, Australia, a remote island in the southwestern Pacific Ocean^73–75^. This research suggests Galapagos shark foraging behaviour can be conditioned by fishing-related practices, however, at Ascension Island, the extent to which these practices drive shark distributions remains unclear, where similar techniques have been used over several decades and longer-term trends in shark abundance and distribution don’t appear to oscillate with recreational fishing intensity^76^. Given that similar events have been documented historically in archival sources, inter-annual variability is more plausibly attributed to natural stochastic variability, rather than recent anthropogenic changes, but warrants further research.

Observed short-term trends may be driven by oceanographic variability, which influences both short-term horizontal and vertical shark distributions, either by affecting prey distributions^77–79^, or the environmental parameters that drive space use^80–82^. For example, Wirsing et al.^77^ found that tiger shark (*Galeocerdo cuvier)* movements were closely tied to prey availability, whilst Papastamatiou et al.^78^ demonstrated how predator space use was determined by optimising foraging strategy for thermal and sensory advantages over prey. In addition, Braun et al.^80^ highlights the importance of mesoscale oceanographic features - such as eddies - in shaping blue shark (*Prionace glauca)* foraging behaviour. Collectively, these studies illustrate that shark distributions are highly responsive to shifting environmental drivers, and thus credit further consideration to determine their role in driving the fluctuations in HSI we report in this study.

At an intra-annual level, results from the remote camera and social media uploads also suggest a degree of seasonality in inshore shark presence, although this was weaker over longer timescales captured by social media data. Peaks were recorded in July - August and February - March, coinciding with the hottest and coolest month for sea surface temperature at Ascension Island, respectively, which may indicate an environmental cause. Seasonal changes in distribution could also be linked to reproductive cycles, as reported in other shark species^83–87^. However, the lack of inter-annual consistency in inshore movements indicates that shark distributions are not driven by seasonality alone, although it is possible that underlying environmental cues are more likely to occur at certain times of the year.

At a finer scale, the high-resolution camera data from the Pierhead reveals a distinct nocturnal rhythm, with a shark peak in observations between dusk and dawn that aligns with recent observations from Norfolk Island^74^, suggesting that Galapagos shark distributions are shaped by predictable diel cycles. The spatiotemporal rhythms documented at Ascension Island may reflect consistent behavioural traits for the species in oceanic island contexts, and be driven by natural foraging patterns, and opportunistic scavenging at coastal landing points^74^. Further research is urgently needed to investigate the influence of climate, oceanography and prey abundance on Galapagos shark movement and the frequency of HSI, particularly given the potential impacts of climate change on species distributions^88^ and HWC^89^.

Qualitative data, such as archival sources and SSI responses, can be vital to understand the evolving nature of HWI^6^ and, here, we show its utility in interpreting observed cycles in shark abundance and behaviours. It can also provide vital detail regarding human sentiment towards HWI, and potentially tracking how, when and why incidents of HWI transform into HWC. Whilst shark presence and activity in Ascension Islands nearshore waters is highly temporally dynamic, it is also punctuated by rapid behavioural and spatial shifts. This dynamic variability is reflected in human experiences, captured through qualitative accounts, which reveal recurrent instances of conflict - such as catch depredation, which aligns with comparative studies that identify depredation as a source of HSI^43,90^; depredation was reported in 214 fisheries between 1979 and 2019 affecting fleets from 44 countries^44^, whilst fear relating to ocean recreation is often a prevalent theme of conflict^37^, despite actual risk of a bite remaining low^33–35^.

Importantly, human sentiment is not fixed; it responds variably to HSI, influenced not only by direct encounters but also by evolving local baselines within a highly transient island population of what is perceived as “normal” shark behaviour^57^. For example, as one Facebook user noted in 2017, “*I have lost thousands of pounds worth of lures and line over the last year but that’s just how it is*”, potentially demonstrating acceptance, or resignation, toward shark-related losses as an embedded feature of life on the Island. By integrating multiple data sources, our approach enables the contextualisation of perceived short-term shifts in HSI - such as those experienced by younger individuals or amongst transient populations - within longer-term oscillations. This is particularly important given that individual perceptions of what constitutes an “acceptable” level of risk play a central role in shaping HWC^5,6^, whilst sentiment is also likely influenced by wider socio-cultural and psychological factors, such as attachment to the ocean and perceptions of management engagement^71^. Further, in a tightly knit community such as Ascension Island, the proximity of social ties to those affected may amplify emotional reactions, leading to sharp, sometimes volatile shifts in public discourse^91^ and management responses^51,92^. Against this backdrop, we also see evidence of ambivalence or positive sentiment in recent decades, potentially indicative of an emerging conservation ethic^93^, or signal the endemic value attributed to sharks as part of Ascension Island’s ecological and cultural identity^42^. More broadly, this complexity reflects patterns observed elsewhere where public sentiment is unaffected, or rebounds after conflict events, though the pace and extent of recovery vary depending on social context and media framing^4,51^. Caution should also be exercised when interpreting shark-related sentiment attached from social media data, as the geographic origin of the poster and degree of connection to local shark interactions can be unclear and may therefore not be wholly representative of local perspectives or experience of HSI.

Whilst many of the methods employed in this study, such as SSIs, archival analysis, and remote camera monitoring, have been previously used on an individual basis to monitor HWI^16,19,22^, this study uniquely combines these approaches to develop a holistic understanding of spatiotemporal variability in HSI. Ascension Island serves as a critical case study to demonstrate that, despite the absence of standardised long-term monitoring, digital and archival data can be triangulated to deepen our understanding of historical baselines of HSI and situate contemporary trends within a broader temporal framework. To our knowledge, the inclusion of publicly available social media uploads represents the first application of such data in this context across any system and demonstrates promising results; temporal patterns in social media activity closely mirrored anecdotal accounts that the 2017 shark incidents were preceded by unusually high inshore activity, highlighting the potential of digital platforms to track temporal trends in HWI. We acknowledge, however, that caution is required when using social media data as a proxy for monitoring the frequency of HSI. For example, temporal trends may reflect fluctuations in the overall volume of uploads (e.g. linked to levels of tourism, posting behaviour or platform uptake), although the latter can be mitigated by the integration of multiple social media channels.

In the current study, desensitisation to inshore shark presence may also have reduced the likelihood of social media uploads over time, even when shark presence remains high, due to a degree of normalisation and posting fatigue. This may explain the relatively low number of social media uploads in July - August 2024, despite high detections of sharks on the pierhead camera at this time, as well as fewer social media uploads posted during the 2021–2022 peaks compared to the 2016 peak, despite the former recording the highest maxN value of 21 individuals. A multimethod approach that integrates data from multiple sources helps to mitigate such limitations and reduce the individual biases inherent in each: archival records may show positivity bias, interviews are subject to recall error, and social media reflects both access and shifting digital norms. By drawing from multiple, temporally and thematically distinct sources, we can more objectively identify points of convergence or contradiction, thereby allowing us to better account for biases within any one dataset. This approach is particularly valuable in marine contexts due to its inherent complexity, less observable nature and the historical emphasis on terrestrial environments, resulting in poorly defined baselines and scarce long-term ecological datasets^94^. As such, a multi-method approach can play a vital role in situating risks in context, and countering the perception that risks associated with HSI are rising^35,95^.

## Concluding remarks

This paper presents a multi-method approach to characterising HSI at a remote oceanic island and outlines a framework broadly applicable for studying HWI across diverse systems and contexts. Our findings reveal both decadal-scale fluctuations and fine-scale spatiotemporal variation in HSI. Interactions are not inherently conflictual; conflict can be influenced as much by shared local narratives, lived experiences and socio-economic factors, and much as ecological drivers, underscoring the importance of long-term monitoring to identify not only *when* interactions occur, but *why* and *how* interactions evolve into conflict. As such, effective management of HSI must move beyond reactive, one-size-fits-all approaches toward more adaptive, context-specific strategies. Monitoring cyclical patterns can support predictive risk management, enabling communities to adjust behaviour during high-risk periods. Recognising these dynamics is essential to fostering coexistence and ensuring long-term support for marine protected areas and broader conservation efforts.

## Materials and methods

### Ascension Island

Ascension Island is the emergent tip of a volcanic peak totalling 88 km^2^ with approximately 800 inhabitants (Fig. 1). There is currently no right to abode on Ascension Island, only those contracted by an employing organisation (and their dependents) may reside on the island, resulting in a highly transient community. Access to the island is restricted to visitors holding a valid entry permit and sponsorship from an existing affiliated entity; as such, tourism is highly limited and has experienced continued declines since COVID-19 and the closure of the airport from 2017 to 2022. Ascension Island’s isolation and small population means its waters have been relatively undisturbed by human activity, including low levels of historic fishing pressure and high abundance of pelagic predators^96^. Sharks have been afforded legal protections within the Ascension Island Exclusive Economic Zone (EEZ) from 2017^97^ Whilst at least 11 shark species are known to inhabit the waters surrounding Ascension Island, two of the most dominant species are Galapagos and silky sharks^98^. In 2019, a marine protected area (MPA) was designated, covering the entirety of the EEZ, totalling 445,000 km^2^, making it the largest MPA in the Atlantic Ocean at the time of designation. The MPA regulations prohibit commercial fishing and mineral extraction, whilst recreational fishing is only permitted within 12 nautical miles of the Island^99^. Recreational boat and shore fishing is common amongst residents^57^, although boat fishing access is limited by the small number of vessels in operation.

Human–shark interactions of Galapagos and silky sharks at Ascension Island were profiled using a multidisciplinary approach incorporating five data sources spanning different spatiotemporal scales:

1. A remote timelapse camera system located at a known conflict hotspot [January 2024–December 2024],
2. Data mining of social media platforms Facebook, Instagram, X (formerly Twitter), and YouTube [2010–2024],
3. Semi-structured interviews with the Island’s current residents [1984–2024],
4. An online survey targeting those currently off-Island, but with previous residency on Ascension Island [1970–2024],
5. Historical literature and archival sources, including language taken from social media comment trails [1684–2016].

### Temporal variation in human–shark interactions

#### Short-term

To investigate short-term (intra-annual) variation in HSI, a remote timelapse camera system (Cam-Do SolarUp: https://cam-do.com/pages/construction-time-lapse-packs) was installed at an elevated position above the water, oriented in a near-vertical, downward-facing position (Fig. 2), and programmed to take still photographs of the inshore waters from the island pierhead in Georgetown (Fig. 1) - a location that had been consistently identified during informal conversations with inhabitants as having periodically high levels of shark activity and associated HSI as a popular shore-fishing location (Clarke, pers. comms., 2024). The camera provided a wide-angle view of the shoreline at the pier, where water depth is relatively shallow (approximately 1–8 m) and water clarity is typically high, allowing for the detection of sharks below the surface. Continuous imaging at this location was also possible beyond daylight hours due to the presence of floodlights at the pierhead. Autonomous timelapse cameras provide a reliable, non-intrusive method for continuous long-term monitoring, allowing for the capture of spatiotemporal patterns that might be missed through traditional observation methods^100,101^. The camera was powered by a 20 W solar panel and programmed to capture a frame at fixed 10-min intervals from 15th January 2024 to 6th December 2024, with occasional temporal gaps in coverage due to technical faults with the camera. Images were manually reviewed to extract the date, time, and the number of sharks visible in each frame. Images with compromised visibility due to surface glare, swell (distortion from wave action), lens obstruction from spray, low-light conditions), and motion blur were excluded from further analysis, yielding 34,810 usable images from a total of 40,157. Intra-annual trends in inshore shark activity were analysed by calculating the maximum number of sharks observed in a single image (MaxN) for each day in the timeseries. Mean daily MaxN was then estimated by modelling daily MaxN as a smooth function of time using a generalised additive model (GAM) with a negative binomial error distribution and log link function. The Negative Binomial family was selected after diagnostic checks revealed overdispersion with a variance to mean ratio of 2.52. We also examined diel variation in inshore shark presence by calculating the proportion of images that contained sharks in each hourly bin across a 24-hour cycle. Hourly probability of presence was then modelled using a binomial GAM including a cubic cyclic smoothing spline of time. All GAMs were fitted using the *mgcv* package in R, version 1.9^102^. Model fit was verified through visual inspection of diagnostic plots (Q-Q plots and residual distributions), which confirmed that models provided a robust fit to the data. All model specifications can be found in SI 1.

#### Medium-term

To investigate medium-term (inter-annual) variation in HSI, we harvested data from four of the largest social media platforms; (1) Facebook, (2) YouTube, (3) X (formerly Twitter), and (4) Instagram (ethical approval granted from the University of Exeter CEC Ethics Committee; application number: 845780). We also ran equivalent searches on TikTok, but returned content was very limited and highly duplicative of posts already sourced on Instagram and Facebook. Due to inherent differences in the functionality of each platform, data collection was tailored depending on accessibility restrictions; however, consistent filtering criteria were applied across platforms to select relevant posts from search results. Specifically, posts were retained for analysis if they included either photographic or video footage of sharks within Ascension Island’s waters, or if the term “shark” was explicitly used within text referring to Ascension Island, and with reference to a specific timeframe (e.g. “*I arrived in 2018 and in my first 1.5 years we rarely seen [sic] sharks. Now we see them daily…”).* Below we detail our approach for each platform.

##### YouTube

We used the YouTube data application programming interface (API), which can return hundreds of relatively consistent search results based on a pre-defined search query^103^. Using the search term “Ascension Island Sharks” we ran the query on a loop with 20 iterations across 5 days, due to daily API quota limitations. Duplicates (based on video title) were removed. A manual inspection of each video was then undertaken and deemed relevant if it pictured a shark within Ascension Island’s nearshore waters (i.e. not including footage from Grattan Seamount, 200 km Southwest of Ascension Island). The YouTube API search was supplemented with a manual scrape, using the same search term (“Ascension Island Sharks”) and subsequently snowballing through the video recommendation list to source relevant content that may not have been retrieved through the API query alone, due to algorithmic biases.

##### X (formerly Twitter)

Due to quota restrictions and prohibitive access costs, we were unable to replicate the automated API-based approach for X (formerly Twitter). A manual search was therefore conducted using the search term: “Ascension Island Sharks”. Returned posts from the search were reviewed individually, and those consisting of hyperlinks to external news articles or alternative social media platforms (such as YouTube videos that had already been captured through previous searches) were excluded from further analysis.

##### Facebook

A manual search was conducted across Facebook. Firstly, the Ascension Island Facebook group (Group ID = 6023925134) was searched using the term “shark”, as this group is the primary, active Facebook group for residents and ex-residents of Ascension Island. This was followed by a more general search across Facebook, with the term “Ascension Island Sharks.”

##### Instagram

Instagram was searched manually using the term “Ascension Island sharks,” in addition to location-based filters to identify uploads taken at Ascension Island. Posts were manually reviewed and included if they featured photographic or video evidence of sharks in the island’s nearshore waters.

**Fig. 6:**
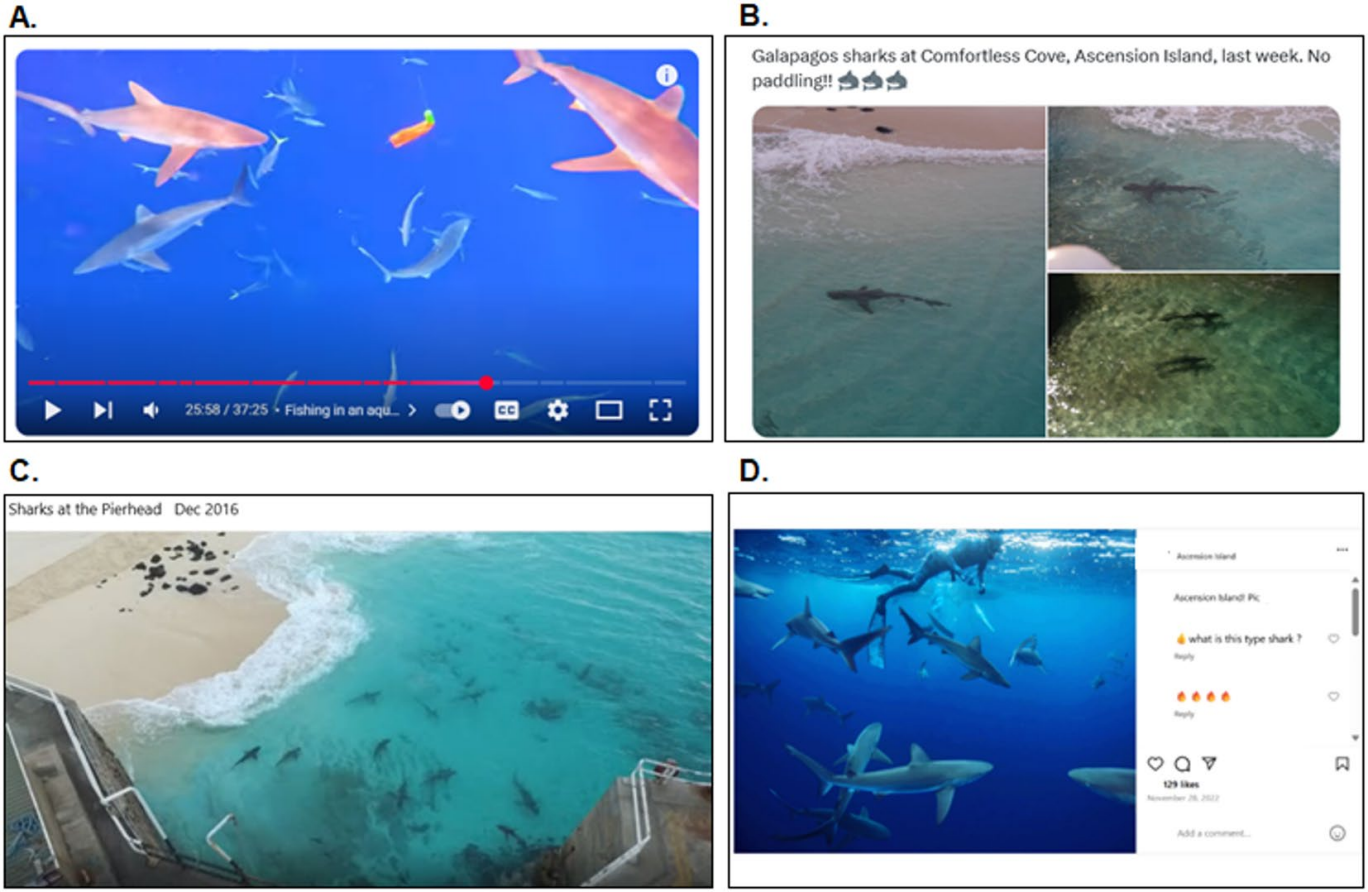
Examples of social media content used to profile spatiotemporal trends of Galapagos and silky sharks within Ascension Island’s nearshore waters from (**A**). YouTube (photo credit: A.Hsieh), (**B**). X (formerly Twitter. photo credit: S.Johnson), (**C**).Facebook (photo credit: J. Roberts-George) and (**D**) Instagram (photo credit: D.Hulme). Any personal identifiers, including social media usernames and profile pictures, have been removed.

All social media posts obtained using methods above were categorised according to whether they included a photo and/or video. For posts that included images (Examples shown in Fig. 6), we extracted the following key variables for analysis; (1) upload date, (2) shark species, (3) number of sharks observed, (4) shark behaviours observed, (5) human activity, (6) an “offshore” vs. “inshore” classification (classified as inshore if the shoreline or ocean floor was visible) and (7) location, if it could be deduced from the coastline or island landmarks, or if the location was given within the associated text (See SI 7 for a breakdown of social media classifications). If a post included an explicit date of recording, this was substituted in place of the upload date for use within time series analysis. No personal details or identifiers from a post were stored for anonymity. As per timelapse camera data, temporal trends in the frequency of social media content containing shark-related photos and videos were analysed using a negative binomial GAM (due to overdispersion within the data) with a log link function fitted to annual total number of posts. To determine the significance of the temporal trend, the full model was compared to an intercept only null model via an analysis of deviance using a ^2^ test which confirmed that the inclusion of the temporal smooth provided a significantly better fit to the data than a model with no temporal predictor. For text-only posts, such as comment threads, the timestamp and any location references (e.g., “*I jumped off the pier in 2001*,* no sharks there then [sic]*”) were logged and used for sentiment analysis.

In addition to social media posts, we also gathered data on the frequency and distribution of shark interactions from island residents through SSIs. Interviews were conducted in-person in April 2024 with current residents of Ascension Island and consisted of 29 open-ended and closed questions in total (SI 8). Given Ascension Island’s small population size, participants were recruited using: (1) purposive sampling, where a researcher approaches individuals based on their suitability and expertise for participation^25^, and (2) snowball sampling, whereby participants are requested to recommend relevant subsequent participants^104^, including at a community event hosted by the research team. Participants were recruited if they satisfied one or more of the following criteria; being a regular ocean user, residing on Ascension Island for a considerable time (> 10 years), or having a key role within the community, such as local business owners or Island councillors. SSI participants had spent on average, 14 ± 14.7 years on Ascension Island (spanning multiple periods for some) and ranged from 4 months to 40 years. Occupations of participants consisted of government officials, key community figures, recreational fishers, contractors and military personnel, with most participants fulfilling multiple sampling criteria (e.g., a recreational fisher who worked for the government).

SSIs were conducted under ethical approvals granted from the ZSL Human Ethics Committee (application number ZSL-HEC-010). All interviews were conducted in accordance with the relevant guidelines and regulations. The purpose of data collection and the expected outputs of the project were outlined prior to participation and verbal consent was obtained from each participant. Participants were also informed that no personal data or identifiable details would be recorded, that data would be securely stored and only named researchers would have access.

#### Long-term

To contextualise data from SSIs across a longer timeframe, we created an online survey to reach prior residents of Ascension Island, with an adapted interview protocol (SI 9). The online survey was broadly analogous in terms of its objectives and content to the SSIs and was developed using SeaSketch software^105^. The survey was disseminated on two Facebook groups that were specifically selected for their connection to past residents of Ascension Island (Group ID 1 = 6023925134, Group ID 2 = 439261969891154) and comprised 15 questions of both open-ended and closed formats. The survey was open for 6 weeks from November - December 2024. Again, the purpose of data collection and expected outputs of the project were outlined and included an informed consent form prior to participation. All methods were approved by the ZSL Human Ethics Committee, application number ZSL-HEC-010.

Archival records and historical literature were searched as part of a broader study on long-term changes to Ascension Island’s marine environment^57^. This included manual searches of the archives on Ascension Island, including the historical records of the local Island newspaper, “The Islander”. Off-Island archives were also searched, including The National Archives of the United Kingdom, The National Scottish Archives, Bodleian Library Archives, The British Library Archives and Manuscripts and The Museum of St Helena (See Burns et al., 2020 for detailed methods). For all the above sources, accounts were sourced dating from the Islands discovery (1501) to the time of data collection (2017). These records were searched for any text references made to abundance within Ascension Island’s marine environment, and included all references made to sharks, given the two recent shark bites at the time of data collection^54,55^. For the purpose of this study, we filtered quotes sourced by Burns et al.^57^ to retain only those that refer explicitly to sharks.

Sample sizes, temporal resolution, and the qualitative nature of textual data sources did not permit a quantitative, year-by-year reconstruction of HSI at Ascension Island over longer (decadal) timescales. As a result, to characterise long-term trends, SSIs, archival sources, online survey responses, and comment trails and textual posts from social media were categorised on an ordinal scale of perceived shark abundance (‘frequently sighted’, ‘occasionally sighted’, or ‘not sighted’ (Table 1) and the relative frequency of each category summarised by decade for the period 1920–2020. Sources were omitted that did not make explicit reference to shark abundance within a designated timeframe. In addition, where a source refers to multiple time periods (e.g. “*Upon arrival I saw a few at a time. The most I saw at first was 3–4. Around April of 2021*,* the population seemed to explode with rarely fewer than a dozen or more appearing near the pier and along the beaches*”), text was divided into discrete time periods for classification.

**Table 1:** Description of classifications of shark abundance criteria based on analysis of social media posts (including comment trails), SSIs, online survey responses, and archival sources.

Classification	Description	Illustrative Quote
Frequently sighted	Text contained words such as “many”, “infested” or “lots” when referring to sharks at Ascension Island.	*“…and this*,* together with the fact that our waters are infested with sharks running to 16 feet might not make trawling a success”*
Occasionally sighted	Text was classified in this category if reference was made to a shark, or an encounter with a shark, without any supplementary information on the level of abundance, or specifically stated that sharks were seen, but not frequently.	“*The very occasional shark was seen underwater but were not interested in swimmers off English bay Deadman’s or Comfortless where people swam and spearfished most days*”
Not sighted	Text explicitly references no sharks being observed.	“*Never saw them there when I was there in the early 90s*”

### The nature of human–shark interactions over time

Whilst the above analyses develop our understanding of how shark abundance, and associated HSI has changed over different temporal scales at Ascension Island, alternative approaches are required to assess how the *nature of interactions* has changed. Here, we utilised text extracted from social media posts and comments, archival records, online surveys and SSIs to explore how the characteristics of HSI has changed over time. Textual data were analysed using a deductive thematic framework analysis supported by NVivo software^106^. Initial data familiarisation identified key themes and sub-themes, which, alongside pre-defined research objectives, informed the construction of a thematic coding framework (SI 5). This iterative process involved iterative refinement of hierarchical codes^25^ with additional themes drawn from existing literature to ensure comprehensive coverage of HIS^32^. Coding was performed until thematic saturation was reached, whereby no new sub-categories or insights emerged from the textual data.

### Impacts of human–shark interactions on human perceptions of sharks

To examine the impact of HSI on human attitudes toward sharks, we employed media discourse analysis using the R package VADER^107^. Automated discourse analysis enables the assessment of large textual datasets, facilitating the identification of temporal patterns and has been previously applied to studies of human–shark conflict in media coverage^46,108,109^. Here, we use automated content analysis to process textual data from social media posts and comment trails, historical archives, SSIs, and short-form responses from our online survey. VADER is a rule-based sentiment analysis tool and lexicon specifically designed for short-form and informal text, such as social media outputs^107^. Additionally, VADER’s functionality accounts for textual nuances, including negations, intensifiers, and punctuation, allowing it to classify entire passages as positive, neutral, or negative using a compound score. The compound score is calculated as a weighted sum of individual word scores, normalised between − 1 (negative) and + 1 (positive) to account for the overall sentiment of the passage, rather than treating words in isolation. This approach enhances contextual accuracy compared to alternative rule-based lexicons that analyse sentiment on a word-by-word basis.

A passage of text is classified as positive if the compound score is larger than, or equal to 0.05, classified as neutral if the compound score falls between − 0.05 and + 0.05, and classified as negative if the score is smaller, or equal to − 0.05. VADER is an efficient, though imperfect, approach to gauge sentiment within the community, over time. As a result, once completed, a manual sense check was conducted on compound scores delivered by the lexicon. As VADER is primarily designed for use on short form and informal text, the output scores were considered sensible and reflective of passages, apart from on textual content from SSIs, which as a result, were subsequently removed from sentiment analysis. All analyses were conducted in R version 4.3.2^110^.

## Supplementary Information

Below is the link to the electronic supplementary material.

Supplementary Material 1

## Data Availability

The datasets used and/or analysed during the current study are available from the corresponding author on reasonable request, with the exception of transcripts from SSIs and outputs from the online survey due to anonymity restrictions and the sensitive nature of the data.

## Declarations

### Statement on inclusion

The majority of the study authors are based in the UK, with two co-authors (Simpson T. and Simpson D.) who live and work at Ascension Island. As permanent residence is not permitted on Ascension Island, inclusion of local authors from the island itself was not feasible. However, we acknowledge that broader representation - particularly from individuals of ethnicities commonly working on the island, such as those from Saint Helena -would have strengthened the perspectives presented. We recognise this as a limitation and are committed to adopting research approaches and processes that incorporate a more diverse range of viewpoints in future work.
