# Supplementary material for: A multi-method approach to characterising dynamic human–shark interactions at a remote oceanic island
Source: Sci Rep. 2026 Apr 13;16:13010. doi: 10.1038/s41598-026-46394-0 (PMC13100140; doi:10.1038/s41598-026-46394-0)
Supplement: Supplementary file 1 — Supplementary Material 1 [file 41598_2026_46394_MOESM1_ESM.docx]

**Supplementary Information 1: Summary of Generalised Additive Model (GAM) specifications and results for trends in human-shark interactions at Ascension Island**

| Model Description | Response | Predictor | Distribution/Link | Deviance explained | edf | Test Statistic | *p*-value |
| --- | --- | --- | --- | --- | --- | --- | --- |
| Intra-annual variation (Piearcam) | Daily MaxN | Date (smooth) | Neg-Binomial/log | 52.1% | 7.72 | 𝜒^2^ = 231.7 | < 0.001 |
| Diel Variation  (Piercam) | Presence/Absence | Hour (with a cyclic cubic spline) | Binomial/logit | 17.2% | 7.25 | 𝜒^2^ = 1027 | <0.001 |
| Seasonality  (Social Media) | Proportion of inshore social media posts | Month (with a cyclic cubic spline) | Binomial/logit | 17.7% | 7.36 | 𝜒^2^ = 19.14 | 0.008 |
| Total Annual Uploads (Social Media) | Total uploads | Year (smooth) | Negative Binomial/log | 64.9% | 3.06 | 𝜒^2^ = 15.63 | 0.003 |
| Annual MaxN  (Social Media) | Annual MaxN | Year (smooth) | Negative Binomial/log | 61.7% | 1.77 | 𝜒^2^ = 17.63 | <0.001 |
| Decadal sightings of "frequently sighted" (Textual data) | Proportion of quotes classified as “frequently sighted” | Decade (linear) | Quasibinomial/logit | 59.9% | 1.00 | *F* = 10.31 | 0.012 |

**Supplementary Information 2: Hourly Distribution of Shark Sightings Detected by the Georgetown Pierhead Camera**


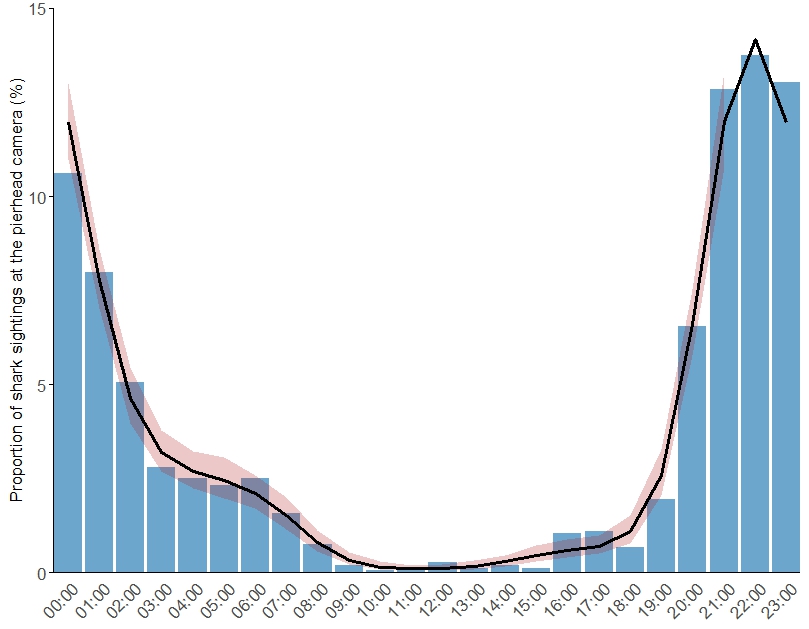


**Supplementary Information 2:** Hourly distribution of shark sightings recorded by the pierhead camera. Bars represent the percentage of total daily sightings observed during each hour, while the black line and shaded ribbon denote the predicted proportions and associated 95% confidence intervals from the fitted generalized additive model (GAM).

##
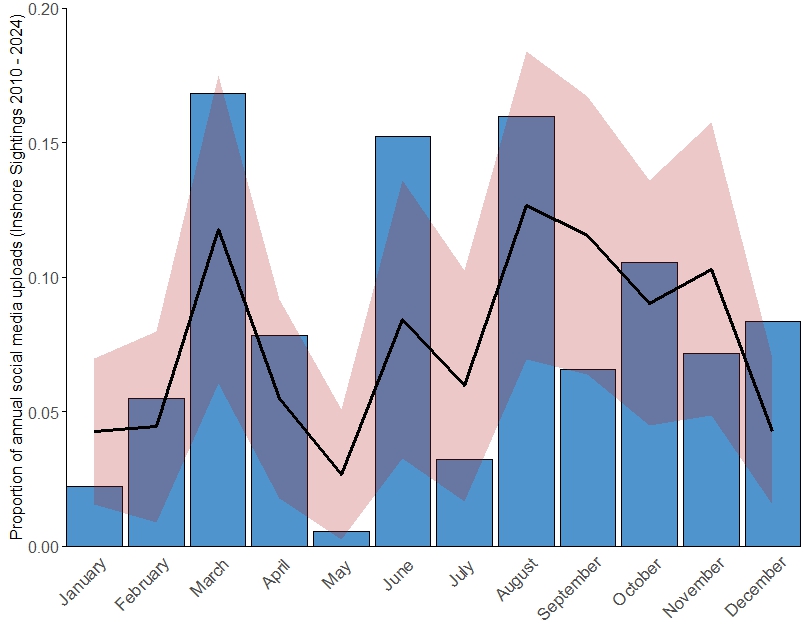
**Supplementary Information 3: Seasonality of inshore social media posts (2010 – 2024)**

**Supplementary Information 3:** Monthly proportion of social media posts featuring photographic or video footage of Galapagos and/or silky sharks at Ascension Island, classified as inshore shark sightings (n=90, 2010 to 2024). Bars represent the average proportion of uploads occurring each month, with the black line and shaded ribbon denote the predicted proportions and 95% confidence intervals.

| Platform | Total number of relevant posts | Number of posts that include a photo or video |
| --- | --- | --- |
| YouTube | 42 (25 sourced through the YouTube API and 17 through manual search) | 42 |
| Facebook | 56 | 49 |
| X (formerly Twitter) | 16 | 13 |
| Instagram | 21 | 21 |
| Comments | 71 (68 from Facebook, 1 from YouTube, 1 from X &1 from Instagram). | 0 |

**Supplementary Information 4: Summary of social media posts and comments sourced from YouTube, Facebook, X and Instagram, 2010 - 2024.**

##
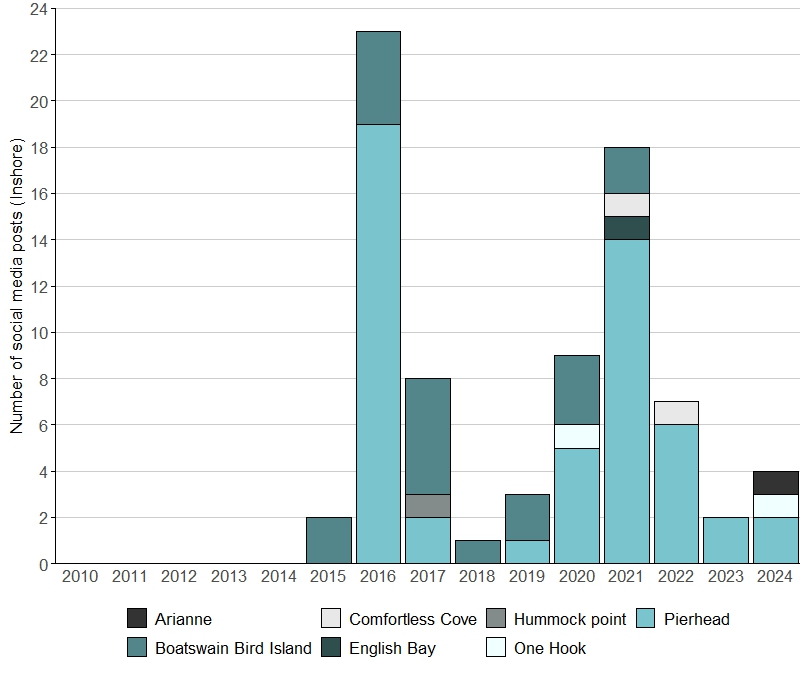
**Supplementary Information 5: Annual Counts of Inshore Social Media Posts by Location (2010–2024)**

**Supplementary Information 5:** Breakdown by location of social media posts categorized as “inshore,” featuring photographic or video footage of Galapagos and/or silky sharks at Ascension Island, for which georeferencing was possible (n = 77; 2010–2024).

## **Supplementary Information 6: Thematic Framework for coding of archive material, interview data, online surveys and social media text**

| Category | Sub-Category | Description | Examples |
| --- | --- | --- | --- |
| Abundance | Frequency | Any description of how often shark sightings, or human-shark interactions were occurring. | “Around April of 2021, the population seemed to explode with rarely fewer than a dozen or more appearing near the pier and along the beaches” |
|  | Regional variation | Any references that have been to spatial features, such as specific named locations with Ascension’s waters, or the North/South/East/West of Ascension or, more generally, inshore/offshore and shallow/deep waters. | “they seemed to prefer deep waters”  “Could be seen reliably at night at the pierhead” |
|  | Age class | Any descriptions of shark presence and/or sightings that make reference to a shark’s size, maturity or presumed age. | “an eighteen foot specimen caught by a fisherman”  “Also caught small juvenile hammerhead off rocks at northeast bay and Ladies loo after dark” |
|  | Intra-annual variability | Any descriptions of the variation of shark sightings or human-shark interactions within a year – including references to the influence of seasonality in presence of sharks. | “While living on the Island fulltime (10-1990 - 1-2001) the sharks would come and leave a few times during the year.”  “I don’t really think it’s a seasonal thing” |
|  | Inter-annual variability | Any reference that is made to the year-to-year variation in shark sightings, or human-shark interactions. | “2012 we didn’t have any sightings and (as MoD employee) we could go snorkelling and people went diving. By the time I came back in 2019 there had been the attacks so MoD banned us from going in the water” |
| Behaviours | Predation | Any mention of a shark “attack”, or of incidents of human predation. Descriptions of attempted, and non-fatal attacks were also coded within this theme. | “attack was expected at any time and beaches were out of bounds”  “It had already been circling us for about 3-4 minutes before it came in for the bump” |
|  | Depredation | Any descriptions of the depredation of fisher catch. This includes any mention of impacts such as lost lures, damage to fishing gear as well as preventative measures adopted and any reference to intra and inter annual variability in observed levels of depredation. | "After losing several sets of fishing tackle to sharks…”  “Now, we have adapted our long-lines so that we tie it to a tube, a car tube (double tube) to prevent sharks from taking the lures” |
|  | Schooling | Any description of aggregations of schooling sharks. This includes references to the size, composition, or frequency of shark groups, as well as observations of behaviours observed | “They are usually found in twos or threes…”  “You can often find 10+ sharks at the pier head of an evening” |
|  | Temperament and condition | Any descriptions that reference the temperament of sharks, either as docile, aggressive, or timid, or that discusses body condition. | “…the sharks appear to be timid…”  “There are few points where Ascension scores high, and that's the docility of the shark”  “Having spent a lot of time here in the water with them I have noticed they are very slender” |

## **Supplementary Information 7: Classification Criteria of Social Media Posts**

| Criteria | Classification |
| --- | --- |
| Upload Date | - Open-ended |
| Filming or Photo Date (if available) | - Open-ended |
| Shark Species | - Open-ended |
| Human Activity | - Swimming - Spearfishing - SCUBA Diving - Snorkelling - Rock Fishing - Boat Fishing - Shoreside (video taken from land) - Unknown |
| Offshore vs Inshore | - Offshore - Inshore (If the shore or coastline is visible) |
| Location | - Open-ended |
| Number of sharks observed | - Open-ended |
| Shark Behaviours | - Swimming - Approaching underwater - Consumption (or attempted) of fish and/or lures at sea - Consumption (or attempted) of fish and/or lures across shallow areas/rocks - Aggressive behaviours towards physical objects - Schooling behaviours |
| Active vs Passive Interaction | - Active (If the shark has approached or made contact - e.g. depredation or approaching underwater) - Passive |

## **Supplementary Information 8: Protocol for semi-structured interviews (SSIs)**

| **No** | **Question** | **Response** |
| --- | --- | --- |
| **Section 1: Socio-demographics** | | |
| 1 | How long have you spent on the island in total, and when? | Open-ended |
| 2 | What ocean-based activities have you done on Ascension (last 5 years)? | - Beach swimming/paddling - Snorkelling - Diving - Boat fishing - Rock fishing - Beach-going - Spearfishing - Other (please describe) |
| 3 | Which of these have you done on Ascension in the last year? | - Beach swimming/paddling - Snorkelling - Diving - Boat fishing - Rock fishing - Beach-going - Spearfishing - Other (please describe) |
| 4 | How often do you tend to do these activities now? | Times per month |
| 5 | How important do you think ocean-based activities are for life on the island? | - Very important - Slightly important - Neutral - Low importance - Not very important |
| 6 | Please provide more detail as to why? | Open-ended |
| **Section 2: Articulating conflict: including understanding dispute level conflicts** | | |
| 7 | How have interactions with sharks changed the way you use the ocean? | Open-ended |
| 8 | What specific impacts have sharks had on your behaviours over the last 5 years? | - I use the ocean less e.g. swimming or paddleboarding - I go fishing less - My enjoyment of using the ocean has deceased - My enjoyment of living on Ascension has been negatively impacted - I have lost money (e.g. through lost lures) - It has stopped me engaging in social occasions or traditions - Other (please specify) |
| 9 | What would you say the biggest impact of shark interactions on Ascension has been? | Open-ended |
| 10 | What % of times do you tend to encounter sharks now? (*question covered for each activity they engage in*) | - Almost always encountered (over 75%) - Frequently encountered (over 50%) - Sometimes encountered (over 25%) - Seldom or never encountered (<5%) |
| 11 | In all your experience of sharks around Ascension, how would you rate current levels of activity? | - Much higher than usual - Slightly higher than usual - About the same - Slightly lower than usual - Much lower than usual |
| 12 | Please provide any further explanation of when shark activity was higher or lower and how that compares to now? | Open-ended |
| 13 | Which of the following behaviours have you experienced sharks doing (*tick all that apply*)? | - Consumption (or attempted) of fish and/or lures at sea - Consumption (or attempted) of fish and/or lures across shallow areas/rocks - Aggressive behaviours towards physical objects, e.g. boats - Schooling behaviours |
| 14 | In your opinion, do anything of the following influence level of interactions (*tick all that apply)?* | - Seasonality (e.g. time of year) - Fishing boat - Gear used - Fishing method (e.g. bait) - Area of fishing |
| 15 | On average, how many lures do you lose per trip? | Open-ended |
| 16 | To what extent to you agree with the following statement, “*sharks compete with fishers for fish*” | - Strongly agree - Slightly agree - Neither agree nor disagree - Slightly disagree - Strongly disagree |
| 17 | To what extent do you agree with the following statement, “*sharks make it unsafe to go in the water in Ascension*” | - Strongly agree - Slightly agree - Neither agree nor disagree - Slightly disagree - Strongly disagree |
| **Section 3: Drivers of conflict: including identifying potential underlying and deep-rooted conflict** | | |
| 18 | To what extent do you agree with the following statement, “*changes in shark population and behaviours is normal for Ascension Island*” | - Strongly agree - Slightly agree - Neither agree nor disagree - Slightly disagree - Strongly disagree |
| 19 | Please identify which, if any, of the following things may have caused a change in shark interactions | - Boat fishing activity including recreational fisheries - Change in populations of other fish - Increase in populations of sharks - Wider oceanographic or climatic changes (e.g. global warming, El Nino) - Conservation measures (e.g. bans on shark fishing or MPA) - Other (please describe) |
| 20 | Of these, in your opinion, which are the most important? | Open-ended |
| 21 | To what extent do you agree with the following statement, “*sharks are important for a healthy marine environment*” | - Strongly agree - Slightly agree - Neither agree nor disagree - Slightly disagree - Strongly disagree |
| 22 | To what extent do you agree with the following statement, “*the Marine Protected Area has increased likelihood of shark interactions*” | - Strongly agree - Slightly agree - Neither agree nor disagree - Slightly disagree - Strongly disagree |
| 23 | To what extent do you agree with the following statement, “*shark populations around Ascension should be managed*” | - Strongly agree - Slightly agree - Neither agree nor disagree - Slightly disagree - Strongly disagree |
| 24 | How do you think shark activity should be managed on Ascension (tick all that apply) | - We should reduce run-off of rubbish or other waste around island - They should be culled - There should be barriers on the beach to prevent shark attacks - They should be protected by law |
| 25 | How do you find out information about sharks on Ascension? | - Social media (e.g. Facebook) - Word of mouth (e.g., friends and family) - Signposts - Events/talks - Newspaper (e.g. Island News) - Websites (e.g. AIG conservation) - Other (please describe) |
| 26 | To what extent do you agree with the following statement, “*I would like more information about sharks on Ascension*” | - Strongly agree - Slightly agree - Neither agree nor disagree - Slightly disagree - Strongly disagree |
| 27 | To what extent do you agree with the following statement, “*I would attend events designed to provide more information around sharks on Ascension*” | - Strongly agree - Slightly agree - Neither agree nor disagree - Slightly disagree - Strongly disagree |
| 28 | Please indicate to what extent physical barriers around Comfortless Cove would increase your enjoyment using the ocean | - Much less enjoyment - Less enjoyment - It would stay the same - More enjoyment - Much more enjoyment |
| 29 | To what extent do you agree with the following statement, “*I feel involved in decisions around shark safety and management*” | - Strongly agree - Slightly agree - Neither agree nor disagree - Slightly disagree - Strongly disagree |

## **Supplementary Information 9: Online survey protocol**

| **No** | **Question** | **Response** |
| --- | --- | --- |
| **Section 1: Socio-demographics** | | |
| 1 | When were you a resident on Ascension Island, and how long for? | Open-ended |
| 2 | What ocean-based activities did you do during your time at Ascension (Chose all that apply) | - Beach swimming/paddling - Snorkelling - Diving - Boat fishing - Rock fishing - Beach-going - Spearfishing - Other (please describe) |
| **Section 2: Participatory Mapping Exercise** | | |
| 3 | Where did you see sharks whilst living on Ascension? (Click on the map to draw) | Open-ended |
| 4 | How often do you encounter sharks at this location? (*question covered for each location identified in question 3*) | - Almost always encountered (over 75%) - Frequently encountered (over 50%) - Sometimes encountered (over 25%) - Seldom or never encountered (<5%) |
| 5 | What type of behaviours have you witnessed at this location? (*question covered for each location identified in question 3*) | - Consumption (or attempted) of fish and/or lures at sea - Consumption (or attempted) of fish and/or lures across shallow areas/rocks - Aggressive behaviours towards physical objects - Schooling behaviours - Approaching underwater - Other (please describe) |
| **Section 3: Articulating and exploring drivers of human-shark interactions** | | |
| 6 | Did you notice any changes in shark behaviour or shark distributions during your time on the island? | Open-ended |
| 7 | To what extent do you agree with the following statement: “Changes in shark populations and behaviours over time is normal on Ascension Island” | - Strongly agree - Slightly agree - Neither agree nor disagree - Slightly disagree - Strongly disagree |
| 8 | Whilst you were living on Ascension, did interactions with sharks change the way you use the ocean? | - Yes - No - I don’t know |
| 9 | Sharks effected my enjoyment of the ocean… | - Positively - Negatively - Neither |
| 10 | What was the impact of sharks on your behaviour (tick all statements that apply) | - I use the ocean less e.g. swimming or paddleboarding - I go fishing less - My enjoyment of using the ocean has deceased - My enjoyment of living on Ascension has been negatively impacted - I have lost money (e.g. through lost lures) - It has stopped me engaging in social occasions or traditions - Other (please specify) |
| 11 | Are there other ways in which sharks affected your life that have not been mentioned? | Open-ended |
| 12 | To what extent do you agree with the following statement: “Sharks made it unsafe to go in the water at Ascension” | - Strongly agree - Slightly agree - Neither agree nor disagree - Slightly disagree - Strongly disagree |
| 13 | In your opinion, do any of the following influence the level of shark interactions? (Chose all that apply) | - Seasonality (e.g time of year) - The type of fishing boat - The type of fishing gear used - The type of fishing method (e.g. bait) - The area of fishing - Other (please describe) |
| 14 | Are there any additional comments you would like to make before ending the survey? | Open-ended |
